# Maternal Smoking During Pregnancy and Adverse Childhood Experiences: The Role of Socioeconomic Status in Adulthood and Perinatal Abuse

**DOI:** 10.1007/s10995-025-04133-3

**Published:** 2025-07-19

**Authors:** Patricia Da Rosa, Ananda Stullich, Matthias Richter

**Affiliations:** 1 Chair of Preventive Pediatrics, TUM School of Medicine and Health, Department of Health and Sport Sciences, Technical University of Munich, Am Olympiacampus 11, TUM Campus im Olympiapark, Munich, 80809 Germany; 2 Chair of Social Determinants of Health, TUM School of Medicine and Health, Department of Health and Sport Sciences, Technical University of Munich, Am Olympiacampus 11, TUM Campus im Olympiapark, Munich, 80809 Germany

**Keywords:** Adverse childhood experiences (ACEs), PRAMS, Pregnancy, Cigarette smoking, Socioeconomic status

## Abstract

**Objectives:**

Previous research has linked adverse childhood experiences (ACEs) to maternal smoking, but the role of socioeconomic status (SES) in adulthood and perinatal abuse remains unclear. This study examined the strength of the association between ACEs and maternal smoking behaviors before, during, and after pregnancy and investigated whether maternal SES and perinatal abuse modify this association.

**Methods:**

This cross-sectional study used data from the Pregnancy Risk Assessment Monitoring System survey (2017–2020). The weighted prevalence of self-reported ACEs and smoking was calculated. Multivariate logistic regression models were conducted to examine the association between ACEs and maternal smoking throughout pregnancy, accounting for maternal SES and perinatal abuse.

**Results:**

Of the 6,595 respondents, nearly 20% of mothers reported experiencing one ACEs, while 29.3% reported three or more. Smoking prevalence was significantly higher among those with ACEs: 2.3% (95% CI 1.7%-3.2%) for mothers reporting zero ACEs, compared to 18.7% (95% CI 16.7%-20.8%) for those reporting three or more ACEs. While SES and perinatal abuse partially explained the association, women with three or more ACEs still had over four times the odds of smoking during pregnancy (aOR = 4.84, 95% CI 3.29–7.10), even after full adjustment.

**Conclusions:**

These results highlight the long-lasting consequences of adversities encountered in early life, which can shape the smoking behaviors of women even during the critical stages of their own pregnancy. There is a need for tobacco control interventions among the most vulnerable that extend beyond the traditional services and address deeply rooted factors from past experiences.

**Supplementary Information:**

The online version contains supplementary material available at 10.1007/s10995-025-04133-3.

## Significance

What Is Already Known on this Subject?

What this Study Adds?

Prior studies have shown that adverse childhood experiences (ACEs) are associated with harmful behaviors, including smoking.

This study examines the association between ACEs and smoking behaviors among pregnant women, demonstrating that ACEs are a significant determinant of maternal smoking. This relationship remains consistent across all three stages of pregnancy, even after accounting for maternal socioeconomic status and perinatal abuse. The findings suggest the importance of trauma-informed smoking cessation strategies, which may be critical for addressing persistent smoking among women with a history of early-life adversity.

**Supplementary Information:**

The online version contains supplementary material available at 10.1007/s10995-025-04133-3.

## Introduction

Cigarette smoking during pregnancy results in significant health risks for both the mother and her child, making it a major concern for U.S. public health (Office of Disease Prevention and Health Promotion, 2023). Although women during pregnancy are more likely to quit smoking than at any other time in their lives (Orleans et al., 2000), cessation remains a challenge. Less than half of the mothers in vulnerable groups, such as women in low socioeconomic conditions, manage to successfully quit during pregnancy (Kipling et al., 2024), highlighting the need for more effective support and interventions during this critical time. Based on the National Center for Health Statistics data on maternal smoking, approximately 170,000 infants in 2021 were exposed to harmful cigarette substances through the placenta (Martin et al., 2023). As with many addictions, smoking often involves a lifelong dependency, underscoring the importance of understanding early-life determinants of maternal smoking and the underlying mechanisms across the life course to effectively prevent and control smoking during pregnancy.

Since the groundbreaking study on Adverse Childhood Experiences (ACEs) by Felitti et al. (1998), research has consistently shown a link between childhood adversity and various adverse health outcomes throughout life. Individuals with a history of ACEs are at greater risk of developing cardiovascular diseases, depression, obesity, cancer, and harmful health behaviors, including addiction to cigarette smoking (Bellis et al., 2019; Felitti et al., 1998; Iob et al., 2020; Lacey et al., 2020; Pear et al., 2017; Petruccelli et al., 2019; Ports et al., 2019). Preventing ACEs could significantly reduce these outcomes, including a 44% decrease in depressive disorders and a 33% reduction in smoking rates (Bellis et al., 2019; Centers for Disease Control and Prevention, 2022).

The potential pathways linking ACEs with maternal smoking can be explained by a complex interplay of biological, material, and psychosocial factors. ACEs encompass both *direct* experiences of abuse or neglect (e.g., physical and sexual abuse) and *indirect* exposure to stressors, such as witnessing domestic violence, living with parents who have mental health issues, or experiencing parental substance abuse or incarceration, potentially leading to an increase in physical and psychological problems later in life (Petruccelli et al., 2019). Chronic early-life stress can lead to neurological changes and dysregulation of the stress response system, increasing susceptibility to addiction as a coping mechanism (Anda et al., 2006). Exposure to ACEs can result in emotional and cognitive impairments, potentially leading to mental health disorders like anxiety and depression, which in turn may drive individuals to engage in high-risk behaviors such as smoking due to its immediate pharmacological benefit resulted from nicotine (Felitti et al., 1998). Additionally, exposure to ACEs often correlates with early smoking initiation and persistent smoking. Research shows the vast majority of current smokers (82.6%) initiated smoking as adolescents or young adults, aged 14 to 25 (GBD 2019 Tobacco Collaborators, 2021). Contributing factors to smoking during pregnancy and early initiation include lack of social support (Masho et al., 2014), discrimination, and parental and peer smoking habits (Joannès et al., 2022). Material pathways include limited financial, and educational resources that can increase stress (Marmot & Wilkinson, 2001) but also reduce access to healthcare, where smoking cessation services and behavior therapy might then be available.

Despite 25 years of research into the link between ACEs and poor health outcomes, there remains a need to understand how early-life adversity translates into persistent health risk behaviors, such as maternal smoking, particularly across critical life stages like pregnancy and postpartum. While previous studies have established that ACEs increase the risk of maternal smoking, the extent to which this relationship is shaped by socioeconomic factors and other stressors in adulthood, such as perinatal abuse, remains unclear (Pear et al., 2017; Swedo et al., 2023). Additionally, despite the well-established importance of pregnancy as a special window of opportunity for cessation (Orleans et al., 2000), little research has examined how ACEs influence smoking behaviors at different time points throughout pregnancy, such as before, during, and after pregnancy. Addressing these gaps is crucial for developing targeted, trauma-informed smoking cessation interventions.

Thus, aiming to understand the mechanisms linking maternal ACEs and smoking in different stages of pregnancy (before, during, and after), this study examines the cumulative role of ACEs as well as both *direct* experiences of child abuse/ neglect and *indirect* exposures to stressful household environments with maternal smoking. The study leverages data from a sample of mothers in two Midwest states in the U.S. who delivered live infants between 2017 and 2020. It investigates the association between ACEs and maternal smoking at different stages of pregnancy and examines the role of maternal socioeconomic status and perinatal abuse in this relationship.

## Methods

### Study Design and Data Source

**Fig. 1:**
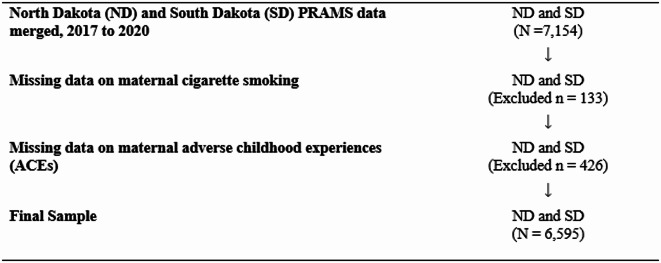
Flowchart of sample selection procedure. Abbreviation: PRAMS, Pregnancy Risk Assessment Monitoring System

This cross-sectional study used a representative sample of mothers who participated in Phase 8 of the Pregnancy Risk Assessment Monitoring System (PRAMS). PRAMS, designed by the Centers for Disease Control and Prevention (CDC), is a population-based surveillance system that gathers data on maternal and infant health outcomes and behaviors. Surveys are disseminated 3–4 months post-delivery. If mothers do not respond to the mailed surveys, they are followed up with a telephone interview. Annually, PRAMS sites typically select between 1,200 and 3,600 mothers for participation (Shulman et al., 2018). Due to the limited availability of data on ACEs, this study only included data from two Midwest states: North Dakota (ND) and South Dakota (SD), which are the only states collecting information on the 10-item ACEs. Figure 1 provides a flow chart of the sample included in this study. The analytical sample included 6,595 participants from the 2017–2020 PRAMS.

### Outcome: Cigarette Smoking

Based on the PRAMS survey, participants self-reported their smoking habits three months before, during the last three months of pregnancy, and three to four months after giving birth. Participants who confirmed smoking any cigarettes in the past two years were asked the following questions: (1) *In the three months before becoming pregnant*,* how many cigarettes did you smoke on an average day?* (2) *During the  last three months of your pregnancy*,* how many cigarettes did you smoke on an average day?* and (3) *How many cigarettes do you currently smoke on an average day?* Respondents could choose from the following options: none, less than 1 cigarette, 1 to 5 cigarettes, 6 to 10 cigarettes, 11 to 20 cigarettes, 21 to 40 cigarettes, and 41 or more. Those who reported smoking at least one cigarette before, during, or after pregnancy were categorized as smokers in each specific period. A new variable, “consistent smoker”, was created to identify mothers who reported smoking consistently across three distinct periods: before, during, and after pregnancy. This variable assigns a value of 1 to respondents who smoked at least one cigarette during each of these periods, indicating consistent smoking behavior. Conversely, mothers who did not meet the smoking criteria for all three stages were assigned a value of 0, indicating no consistent smoking across the stages.

### Exposure: Adverse Childhood Experiences (ACEs)

ACEs were measured based on the 10-item Adverse Childhood Experiences questionnaire (refer to Appendix A for details) collected in the ND and SD PRAMS survey. First, a cumulative ACEs score was computed. This score was then categorized into zero, 1, 2, and 3+. The cutoff point of three or more ACEs was established to create a more conservative measure due to the sensitive nature of the questions involved (Joannès et al., 2022). Additionally, to distinguish between *direct* (child abuse/ neglect) and *indirect* stressors (stressful household environments), the 10-item questionnaire was divided into two categories: stressful household environments and child abuse/neglect. For more details, please refer to Appendix B.

### Mediating Factors

Socioeconomic Status in adulthood: Maternal education was grouped into four levels, including less than high school, high school graduate, some college, and college graduate or higher. Household income was categorized as ≤ $24,000, $24,001-$60,000, $60,001-$85,000, and above $85,001. Due to the small number in the original categories, health insurance during prenatal care was dichotomized into private/other and Medicaid/uninsured.

To account for maternal experiences of abuse in adulthood, the variable “perinatal abuse” was created. This variable measures maternal experiences of physical, sexual, and psychological violence within the 12 months before or during pregnancy by her husband, ex-husband, or someone else. It is based on six specific questions from PRAMS, which inquire about physical and sexual violence by a husband, ex-husband, or another individual. Appendix C provides the specific questions used in this measurement of perinatal abuse. By summing the number of instances of abuse endured in the 12 months preceding pregnancy or during pregnancy, a total score variable was obtained. This score was then dichotomized into ‘no abuse’ and ‘at least one instance of abuse.’

### Covariables

Covariables included self-reported information. Demographic factors: Maternal age was grouped into four categories: under 24, 25–29, 30–34, and 35 years and above. Maternal race and ethnicity were classified as White (non-Hispanic), American Indian (non-Hispanic), and other categories to reflect the ND and SD population demographics. Marital status was measured as married or not married (other).

The number of previous births was grouped either as 0, 1, 2, or more than 3. Additionally, the specific year of the infant’s birth and state residence were added to the analytical models to account for changes in smoking prevalence over time and state tobacco control policies. Although depression is a risk factor for smoking, it is also associated with ACEs thus, an intermediate factor, the variable ‘depression’ was not included in the model to avoid over-adjustment.

### Statistical Analysis

Descriptive statistics, including weighted frequencies and percentages, were calculated to summarize the distribution of key variables, such as ACEs, smoking status, and sociodemographic characteristics. Second, bivariate associations were carried out using chi-square tests to compare the prevalence of smoking at the three stages (before, during, and after) of pregnancy across different ACEs scores, as well as the number of stressful household environments and child abuse/neglect. Finally, multivariate logistic regressions were performed to estimate the odds ratios (OR) and 95% Confidence Intervals (CI) for smoking at the three stages. These regression models compared mothers with one, two, and three or more ACEs to those with none (Model 1). The models were initially controlled for previously reported confounders and demographic covariates (Model 2) and subsequently adjusted to identify potential mediators, including maternal socioeconomic status (Model 3) and perinatal abuse (Model 4). Similar analytical models were applied to household stressors and childhood abuse/neglect to determine their relationship with maternal smoking. The four models are described below.

#### Model 1

This model was a bivariate analysis designed to identify the crude association between ACEs and maternal smoking.

#### Model 2

This model was built upon Model 1 by incorporating demographic variables such as maternal age, race/ethnicity, marital status, and year of birth.

#### Model 3

This model included variables from Model 2 and added maternal socioeconomic status, such as maternal education and health insurance during pregnancy.

#### Model 4

The fully adjusted model included all the variables from Model 3 and additionally added information on perinatal abuse experience before/during pregnancy.

Only participants with complete data on ACEs and smoking were included in the analysis. Covariables, including race and health insurance, had approximately 5% missing data. Thus, a category for these missing values was created and incorporated into the models. All analyses were conducted using the Complex Sample Module in IBM SPSS version 29.0 (IBM Corp, 2022) to appropriately account for PRAMS survey design and non-response rate. Statistically significant results were defined as those with a p-value less than 0.05.

### Ethical Approval

The study used de-identified data, exempting it from the federal definition of human subject research (Code of Federal Regulations, 45 CFR 46.102) and the University Institutional Review Board review. However, the use of this data was approved by the CDC PRAMS working sites.

## Results

Among the 7,154 women who participated in the 2017–2020 PRAMS survey, 133 were excluded due to missing data on smoking, and 426 were excluded due to missing information on ACEs. This resulted in a final sample of 6,595 participants (weighted population: 78,768), representing 92.2% of the total sample (Fig. 1). In the study population, approximately 64.9% of mothers were aged 25–34, while 22.3% were 24 or younger. The majority were non-Hispanic White (73.0%), married (66.9%), and had private insurance (66.0%). The aggregated weighted prevalence of cigarette smoking before, during, and after pregnancy was 21.1%, 9.3%, and 13.8%, respectively. Nearly half of the mothers who smoked before pregnancy continued to smoke during and after pregnancy. The prevalence of consistent smoking throughout these periods was 8.5%. The most commonly reported ACE was parental separation or divorce before the age of 18, at 39.2%, followed by household substance abuse at 27.9%. The least reported ACE was physical neglect, at 6.4%. Approximately one-third of the mothers reported experiencing 2–5 forms of household stressors, and 21.1% reported experiencing 2–5 forms of child abuse/neglect (Table 1).

**Table 1:** Sample characteristics, ND and SD, PRAMS, 2017–2020

Characteristic	Levels	No. of respondents (*N*)*	Weighted percent (%)
Cigarette smoking			
	Before pregnancy	1775	21.1
	During pregnancy	783	9.3
	After pregnancy	1181	13.8
	Consistent smoker	708	8.5
Number of ACEs	None	2332	39.9
	1	1326	19.6
	2	750	11.2
	3 or more	2187	29.3
ACEs- item			
	Parental separation/divorce	2938	39.2
	Household substance abuse	2097	27.9
	Household member mental illness	1681	25.1
	Verbal abuse	1739	24.8
	Physical abuse	1200	16.0
	Emotional neglect	1206	15.7
	Sexual abuse	1044	13.5
	Household violence against mother	983	12.2
	Parental incarceration	854	9.8
	Physical neglect	530	6.4
Household stressors			
	None	2676	45.4
	1 form	1593	23.8
	2–5 forms	2326	30.9
Childhood abuse/neglect			
	None	4103	65.1
	1 form	916	13.8
	2–5 forms	1576	21.1
Perinatal abuse			
	None	6184	95.8
	At least one	411	4.2
Maternal age			
	< 24	1714	22.3
	25–29	2206	34.6
	30–34	1866	30.3
	35+	809	12.8
Maternal race/ethnicity			
	White, non-Hispanic	3056	73.0
	American Indian, non-Hispanic	1874	10.1
	Other	1634	16.4
	Missing	31	0.6
Marital status			
	Married	3746	66.9
	Other	2849	33.1
Maternal education			
	Less than high school	1039	10.4
	High school	1492	21.9
	Some college	2021	29.5
	College graduate or more	2000	37.6
	Missing	43	0.6
Household income	<= $24,000	2268	24.1
	$24,001- $60,000	1803	28.7
	$60,001- $85,000	754	14.7
	$85,001 +	1354	27.6
	Missing	416	4.9
Insurance during prenatal care			
	Private/Other	3492	66.0
	Medicaid/uninsured	2679	29.4
	Missing	424	4.6
Number previous birth			
	None	2127	34.9
	1	2001	32.1
	2	1241	18.4
	3 or more	1225	14.7
	Missing	1	
State			
	North Dakota (ND)	2798	48.3
	South Dakota (SD)	3797	51.7
Period (infant’s year of birth)			
	2017	1588	25.9
	2018	1768	25.7
	2019	1631	24.7
	2020	1608	23.7

*Unweighted N

Abbreviation: ACEs, Adverse Childhood Experiences

**Fig. 2:**
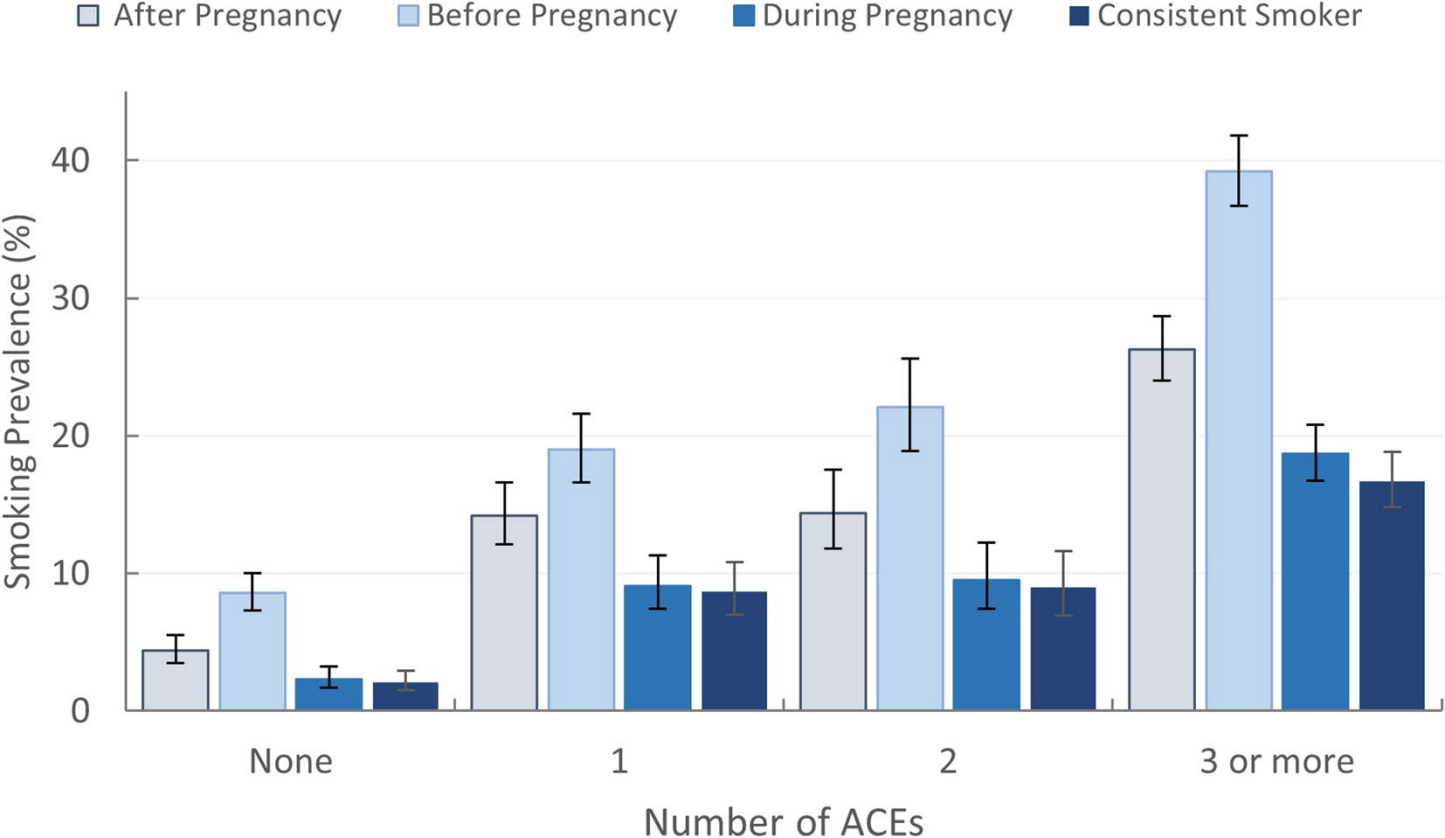
Prevalence of smoking before, during, and after pregnancy by the number of ACEs, ND and SD, PRAMS, 2017-2020

Figure 2 presents the prevalence of smoking before, during, and after pregnancy, and consistent smoking categorized by the number of ACEs. Bivariate analysis indicated a clear gradient. Mothers exposed to more adverse experiences were more likely to smoke before, during, and after pregnancy. For instance, mothers reporting zero ACEs had a smoking prevalence of 8.6% before pregnancy, whereas those exposed to three or more ACEs had a significantly higher prevalence at 39.2%. Table 2 also presents the weighted prevalence of smoking for all three stages, categorized by stressful household environments and child abuse/neglect.

**Table 2:** Prevalence of maternal smoking before, during and after pregnancy by the number of aces and number of households dysfunction and childhood abuse, ND and SD, PRAMS, 2017–2020

Variables	Unweighted	Prevalence Before Pregnancy	Prevalence During Pregnancy	Prevalence After Pregnancy
	*N* = 6,595	% (95%CI)*	% (95%CI)*	% (95%CI)*
**Number of ACEs**				
None	2,332	8.6 (7.3–10.0)	2.3 (1.7–3.2)	4.4 (3.5–5.5)
1	1,326	19.0 (16.6–21.6)	9.1 (7.4–11.3)	14.2 (12.1–16.6)
2	750	22.1 (18.9–25.6)	9.5 (7.4–12.2)	14.4 (11.8–17.5)
3 or more	2,187	39.2 (36.7–41.8)	18.7 (16.7–20.8)	26.3 (24.0-28.7)
**Household stressors**				
None	2,676	9.4 (8.2–10.7)	2.7 (2.1–3.5)	4.9 (4.0-5.9)
1	1,593	22.1 (19.8–24.6)	10.0 (8.4–12.0)	15.9 (13.9–18.2)
2–5	2,326	37.6 (35.1–40.1)	18.3 (16.3–20.3)	25.4 (23.3–27.7)
**Childhood abuse/neglect**				
None	4,103	14.1 (12.9–15.4)	5.6 (4.8–6.5)	9.1 (8.1–10.2)
1	916	26.1 (22.9–29.5)	11.1 (8.9–13.6)	17.1 (14.4–20.1)
2–5	1,576	39.4 (36.4–42.5)	19.3 (16.9–21.9)	26.3 (23.7–29.2)

Note. Rao-Scott adjusted chi-square statistic *P* <.001.

*Weighted prevalence

Abbreviations: ACEs, Adverse Childhood Experiences; CI, confidence interval

Results from logistic regression models with smoking before, during, and after pregnancy as the outcome and ACEs as the independent variable consistently showed that participants with higher ACEs were more likely to smoke, after adjusting for confounders and mediators. For instance, in Model 1, mothers reporting the highest ACEs (3 or more) had a crude OR of 6.89 (95% CI 5.63–8.43) compared to mothers without ACEs (Table 3). In Model 2, after adjusting for demographic factors (maternal age, race/ethnicity, marital status, and year of delivery), the OR was reduced to 4.32 (95% CI 3.48–5.36). In model 3, when adjusting for socioeconomic status measures, the OR reduces further to 3.59 (95% CI 2.87–4.48) and then later to 3.43 (95% CI 2.74–4.29), when perinatal abuse was included in the model. These results indicate that the mothers with exposure to at least three ACEs were more than three times as likely to smoke before pregnancy compared to mothers with no ACEs. Similar findings were found for smoking during (Table 4), after pregnancy (Table 5), and among consistent smokers (data not shown). Similar results were also observed for cumulative exposure to stressful household environments and child abuse/neglect. However, household stressors appear to be more strongly associated with maternal smoking than childhood abuse. For instance, during pregnancy, mothers experiencing multiple household stressors (2–5) had an aOR of 3.30 (2.29–4.75), while those with 2–5 experiences of childhood abuse had an aOR of 1.50 (1.14–1.98).

**Table 3:** Association between categories of adverse childhood experiences (ACEs) and the likelihood of maternal smoking before pregnancy ND and SD, PRAMS, 2017–2020 (*N* = 6,595)

Variables	Model 1 Unadjusted OR (95% CI)	Model 2 aOR (95% CI)	Model 3 aOR (95% CI)	Model 4 aOR (95% CI)
**Number of ACEs**				
None	Ref.	Ref.	Ref.	Ref.
1	2.50 (1.98–3.64)	1.97 (1.53–2.52)	1.84 (1.43–2.38)	1.84 (1.43–2.37)
2	3.02 (2.33–3.92)	2.24 (1.70–2.95)	2.01 (1.52–2.68)	1.98 (1.49–2.64)
3 or more	6.89 (5.63–8.43)	4.32 (3.48–5.36)	3.59 (2.87–4.48)	3.43 (2.74–4.29)
**Household stressors**				
None	Ref.	Ref.		Ref.
1	2.46 (1.99–3.04)	1.88 (1.50–2.35)	1.76 (1.40–2.21)	1.76 (1.40–2.21)
2–5	4.07 (3.31–5.02)	2.55 (2.03–3.20)	2.23 (1.76–2.83)	2.21 (1.75–2.80)
**Childhood abuse/neglect**				
None	Ref.	Ref.	Ref.	Ref.
1	1.40 (1.14–1.74)	1.51 (1.20–1.89)	1.44 (1.14–1.83)	1.41 (1.11–1.80)
2–5	1.97 (1.63–2.39)	1.95 (1.59–2.40)	1.77 (1.42–2.20)	1.69 (1.35–2.11)

Model 1: Aces and smoking before pregnancy

Model 2: Model 1 + age + race/ethnicity + marital status + infant’s year of birth

Model 3: Model 1 + Model 2 + socioeconomic (maternal education + insurance)

Model 4: Model 1 + Model 2 + Model 3 + perinatal abuse

Abbreviations: CI, confidence interval; aOR, adjusted odds ratio

**Table 4:** Association between categories of adverse childhood experiences (ACEs) and the likelihood of maternal smoking during pregnancy, ND and SD, PRAMS, 2017–2020 (*N* = 6,595)

Variables	Model 1 Unadjusted OR (95% CI)	Model 2 aOR (95% CI)	Model 3 aOR (95% CI)	Model 4 aOR (95% CI)
**Number of ACEs**				
None	Ref.	Ref.	Ref.	Ref.
1	4.23 (2.85–6.27)	3.39 (2.26–5.08)	3.21 (2.12–4.86)	3.20 (2.12–4.84)
2	4.43 (2.91–6.76)	3.42 (2.23–5.26)	3.08 (1.97–4.80)	3.04 (1.94–4.74)
3 or more	9.64 (6.81–13.65)	6.14 (4.24–8.90)	5.08 (3.47–7.43)	4.84 (3.29–7.10)
**Household stressors**				
None	Ref.	Ref.		Ref.
1	3.63 (2.58–5.12)	2.87 (2.02–4.01)	2.65 (1.85–3.79)	2.65 (1.85–3.80)
2–5	5.79 (4.17–8.03)	3.84 (2.70–5.45)	3.33 (2.31–4.79)	3.30 (2.29–4.75)
**Childhood abuse**				
None	Ref.	Ref.	Ref.	Ref.
1	1.26 (0.93–1.70)	1.34 (0.98–1.81)	1.29 (0.94–1.78)	1.26 (0.91–1.73)
2–5	1.83 (1.43–2.35)	1.76 (1.36–2.28)	1.58 (1.20–2.08)	1.50 (1.14–1.98)

Model 1: Aces and smoking before pregnancy

Model 2: Model 1 + age + race/ethnicity + marital status + infant’s year of birth

Model 3: Model 1 + Model 2 + socioeconomic (maternal education + insurance)

Model 4: Model 1 + Model 2 + Model 3 + perinatal abuse

Abbreviations: CI, confidence interval; aOR, adjusted odds ratio

**Table 5:** Association between categories of adverse childhood experiences (ACEs) and the likelihood of maternal smoking after pregnancy, ND and SD, PRAMS, 2017–2020 (*N* = 6,595)

Variables	Model 1 Unadjusted OR (95% CI)	Model 2 aOR (95% CI)	Model 3 aOR (95% CI)	Model 4 aOR (95% CI)
Number of ACEs				
None	Ref.	Ref.	Ref.	Ref.
1	3.62 (2.69–4.88)	2.84 (2.09–3.87)	2.72 (1.98–3.73)	2.71 (1.98–3.72)
2	3.68 (2.65–5.10)	2.74 (1.95–3.84)	2.49 (1.76–3.51)	2.46 (1.74–3.48)
3 or more	7.78 (5.99–10.12)	4.72 (3.56–6.26)	3.93 (2.95–5.25)	3.79 (2.83–5.06)
**Household stressors**				
None	Ref.	Ref.		Ref.
1	3.37 (2.58–4.40)	2.58 (1.96–3.40)	2.42 (1.82–3.22)	2.43 (1.83–3.22)
2–5	4.97 (3.83–6.45)	3.10 (2.34–4.11)	2.71 (2.03–3.63)	2.70 (2.07–3.61)
**Childhood abuse/neglect**				
None	Ref.	Ref.	Ref.	Ref.
1	1.28 (1.00-1.64)	1.35 (1.04–1.76)	1.31 (1.00-1.73)	1.29 (0.98–1.69)
2–5	1.71 (1.38–2.13)	1.65 (1.31–2.08)	1.49 (1.17–1.89)	1.43 (1.12–1.82)

Model 1: Aces and smoking before pregnancy

Model 2: Model 1 + age + race/ethnicity + marital status + infant’s year of birth

Model 3: Model 1 + Model 2 + socioeconomic (maternal education + insurance)

Model 4: Model 1 + Model 2 + Model 3 + perinatal abuse

Abbreviations: CI, confidence interval; aOR, adjusted odds ratio

The number of previous births and state of residency were not statistically significant and were subsequently removed from the final models. Household income was highly correlated with maternal education (*r* =.61); therefore, only maternal education was included in the models.

## Discussion

Using data from a population-based survey (PRAMS), nearly one in 10 mothers reported smoking during pregnancy, and 13.8% after pregnancy. An estimated 60% had experienced at least one ACE before age 18, with 30% reporting experiencing three or more ACEs. These estimates are consistent with previous studies (Centers for Disease Control and Prevention, 2022; Swedo et al., 2023) and underscore the high prevalence of trauma in this population.

Moreover, we found that mothers with a history of childhood adversity were significantly more likely to smoke before, during, and after pregnancy, adding to the growing existing evidence that early trauma influences maternal smoking behaviors (Pear et al., 2017; Young-Wolff et al., 2019). Mothers who reported three or more ACEs were found to be nearly five times more likely to smoke during pregnancy and maternal SES and perinatal abuse only partially explained this association. Similarly, Pear et al. (2017), using the U.S. National Longitudinal Survey of Youth, found that mothers exposed to physical abuse in childhood were 39% more likely to smoke during pregnancy. Our study extends these findings by also examining sustained smoking patterns across pregnancy stages, emphasizing the long-term impact of childhood trauma on maternal smoking trajectories, and the role of maternal SES and perinatal abuse.

Additionally, in our study, stressful household environments appear to be a stronger predictor of smoking during and after pregnancy than direct child abuse or neglect. This may be due to lower reported rates of abuse and neglect or because household stressors tend to be more constant and intense, thereby having a greater impact on smoking behavior later in life. However, these findings highlight the need for further research into how different early-life adversity affects health behaviors.

The potential pathways that link ACEs to smoking behavior are complex and may include both direct and indirect influences across the life course. Direct or biological pathways such as genetic predispositions, chronic stress response dysregulation, and inflammation, along with changes in metabolism during pregnancy, may increase vulnerability to addiction (Iob et al., 2020; Scherman et al., 2018; Silventoinen et al., 2022). Indirectly, ACEs can contribute to mental health outcomes such as depression, anxiety, and post-traumatic stress disorder, conditions often associated with smoking as a coping mechanism (Kappel et al., 2021; Pear et al., 2017; Petruccelli et al., 2019; Puetz & McCrory, 2015). Psychosocial stress and lack of social support may further increase the likelihood of smoking (Goodwin et al., 2017; Stubbs et al., 2019). From a material standpoint, low SES and chronic stress in adulthood may drive the use of substances like tobacco as coping strategies.

In this context, future research should explore how resilience, childhood SES, and social support may shape the relationship between ACEs and smoking among pregnant women. Longitudinal studies are especially needed to examine the persistence of these associations, which could lead to more effective and lasting public health interventions. In addition, mixed-methods research should be considered. Quantitative analysis can capture the strength of the associations, while qualitative insights can illuminate mothers’ lived experiences, reasons for smoking, and challenges to quitting. This integrated approach would offer a fuller understanding of how early-life adversity translates into maternal smoking behaviors (Tariq & Woodman, 2013).

While more research is needed to understand all contributing factors, this study highlights the importance of integrating trauma-informed care (TIC) into smoking cessation and healthcare programs. Tailoring services to the unique needs of women affected by trauma across the reproductive continuum, including before, during, and after pregnancy, can help reduce smoking prevalence and reduce its harmful effects on both mother and child. Strengthening connections between mental health services and prenatal care is crucial, especially for pregnant women facing high stress or increased vulnerability. Ensuring these services are accessible and non-stigmatizing can enhance engagement with both mental health and smoking cessation support.

However, effectively implementing trauma-informed care requires more than a patient-centered approach. It calls for a shift in healthcare culture, creating safe, supportive environments that recognize and respond to trauma-related triggers associated with cigarette smoking (Substance Abuse and Mental Health Services Administration, 2014). Embedding TIC into prenatal and cessation programs enables ethical, sustainable, and comprehensive support for pregnant women seeking to quit smoking while coping with the psychological impacts of trauma.

Finally, this does not suggest that pregnant women should be routinely screened for ACEs. Rather, it supports a more holistic approach to prevention and intervention that integrates Quitlines and other traditional strategies with a broader, more coordinated framework. A best-practice model for supporting pregnant women with high ACE scores would include trauma-informed care, comprehensive support services, and continuous monitoring (Jones et al., 2020). Ongoing training for healthcare providers on the impacts of ACEs, combined with integrated care models that bring together maternity care, mental health, and smoking cessation services, is essential for delivering coordinated and compassionate care.

### Strengths and Limitations

The strengths of our study include large sample size and comprehensive data on ACEs, maternal smoking at different stages of pregnancy, and key covariates while using a standardized protocol. Nevertheless, this study also has limitations. First, as a cross-sectional study, causality cannot be established. Second, reliance on self-reported data for smoking and ACEs may lead to misclassification and underestimation due to social desirability and recall biases. Third, the ACEs measure does not capture the frequency, severity, or full range of negative childhood experiences. Fourth, the strength of the observed associations could be overestimated by residual confounding such as maternal resilience, conscientiousness, or social support (Joannès et al., 2022). Finally, since data come from two primarily rural states with limited racial diversity and a high proportion of privately insured mothers, the findings may not be generalizable to more diverse or urban populations.

## Conclusion

Leveraging data from the 2017–2020 PRAMS survey, this study found a significant dose-response association between ACEs and maternal smoking. Additionally, maternal socioeconomic status and perinatal abuse emerged as potential mediators in this association. These findings highlight the complex interplay between early-life adversity and maternal smoking behaviors.

## Electronic Supplementary Material

Below is the link to the electronic supplementary material.

Supplementary Material 1

## Data Availability

All data is publicly accessible from the Centers for Disease Control and Prevention.
