# Supplementary material for: Maternal Smoking During Pregnancy and Adverse Childhood Experiences: The Role of Socioeconomic Status in Adulthood and Perinatal Abuse
Source: Matern Child Health J. 2025 Jul 19;29(9):1334–44. doi: 10.1007/s10995-025-04133-3 (PMC12460459; doi:10.1007/s10995-025-04133-3)
Supplement: Supplementary file 1 — Supplementary Material 1 [file 10995_2025_4133_MOESM1_ESM.docx]

**Appendix A**

Questions on Adverse Childhood Experience (ACE), 10-item, PRAMS, 2017-2020

| **No. items** | **Theme** | **Question** | **Levels** |
| --- | --- | --- | --- |
| 1 | Parental separation or divorced | Were your parents ever separated or divorced? | 1= NO  2= YES |
| 2 | Household member substance abuse | Did you live with anyone who was a problem drinker or alcoholic or who used street drugs? | 1= NO  2= YES |
| 3 | Household member mental Illness | Was a household member depressed or mentally ill, or did a household member attempt suicide? | 1= NO  2= YES |
| 4 | Incarceration household member | Did a household member go to prison? | 1= NO  2= YES |
| 5 | Household violence against mother | Was your mother or stepmother pushed, grabbed, slapped, or had something thrown at her OR sometimes, often, or very often kicked, bitten, hit with a fist, or hit with something hard OR ever repeatedly hit at least a few minutes or threatened with a gun or knife? | 1= NO  2= YES |
| 6 | Sexual abuse | Did an adult or person at least 5 years older than you ever touch or fondle you or have you touch their body in a sexual way OR attempt or actually have oral, anal, or vaginal intercourse with you? | 1= NO  2= YES |
| 7 | Emotional/verbal abuse | Did a parent or other adult in the household swear at you, insult you, put you down, or humiliate you OR act in a way that made you afraid that you might be physically hurt? | 1= NO  2= YES |
| 8 | Physical abuse | Did a parent or other adult in the household push, grab, slap, or throw something at you OR ever hit you so hard that you had marks or were injured? | 1= NO  2= YES |
| 9 | Emotional neglect | Did you feel that no one in your family loved you or thought you were important or special OR your family didn’t look out for each other, feel close to each other, or support each other? | 1= NO  2= YES |
| 10 | Physical neglect | Did you feel that you didn’t have enough to eat, had to wear dirty clothes, and had no one to protect you OR your parents were too drunk or high to take care of you or take you to the doctor if you needed it? | 1= NO  2= YES |

**Appendix B**

Questions on indirect (household stressors) and direct (childhood abuse/neglect) stressors in childhood, PRAMS, 2017-2020

| **Household stressors** | | |
| --- | --- | --- |
| **Theme** | **Question** | **Levels** |
| Parental separation or divorced | Were your parents ever separated or divorced? | 1= NO  2= YES |
| Household member substance abuse | Did you live with anyone who was a problem drinker or alcoholic or who used street drugs? | 1= NO  2= YES |
| Household member mental Illness | Was a household member depressed or mentally ill, or did a household member attempt suicide? | 1= NO  2= YES |
| Incarceration household member | Did a household member go to prison? | 1= NO  2= YES |
| Household violence against mother | Was your mother or stepmother pushed, grabbed, slapped, or had something thrown at her OR sometimes, often, or very often kicked, bitten, hit with a fist, or hit with something hard OR ever repeatedly hit at least a few minutes or threatened with a gun or knife? | 1= NO  2= YES |
| **Childhood abuse/neglect** | |  |
| **Theme** | **Question** | **Levels** |
| Sexual abuse | Did an adult or person at least 5 years older than you ever touch or fondle you or have you touch their body in a sexual way OR attempt or actually have oral, anal, or vaginal intercourse with you? | 1= NO  2= YES |
| Emotional/verbal abuse | Did a parent or other adult in the household swear at you, insult you, put you down, or humiliate you OR act in a way that made you afraid that you might be physically hurt? | 1= NO  2= YES |
| Physical abuse | Did a parent or other adult in the household push, grab, slap, or throw something at you OR ever hit you so hard that you had marks or were injured? | 1= NO  2= YES |
| Emotional neglect | Did you feel that no one in your family loved you or thought you were important or special OR your family didn’t look out for each other, feel close to each other, or support each other? | 1= NO  2= YES |
| Physical neglect | Did you feel that you didn’t have enough to eat, had to wear dirty clothes, and had no one to protect you OR your parents were too drunk or high to take care of you or take you to the doctor if you needed it? | 1= NO  2= YES |

# Appendix C

Questions on perinatal abuse before or during pregnancy, PRAMS, 2017-2020

| **Variable name** | **Question** | **Levels** |
| --- | --- | --- |
| PAB6HUS | In the 12 months before you got pregnant with your new baby, did any of the following people push, hit, slap, kick, choke, or physically hurt you in any other way? For each person, check No if they did not hurt you during this time or yes if they did. My husband or partner | 1= NO  2= YES |
| PAB_XHUS | In the 12 months before you got pregnant with your new baby, did any of the following people push, hit, slap, kick, choke, or physically hurt you in any other way? For each person, check No if they did not hurt you during this time or yes if they did. My ex-husband or ex-partner | 1= NO  2= YES |
| PAB_OTH | In the 12 months before you got pregnant with your new baby, did any of the following people push, hit, slap, kick, choke, or physically hurt you in any other way? For each person, check No if they did not hurt you during this time or yes if they did. Someone else | 1= NO  2= YES |
| PAD6HUS | During your most recent pregnancy, did any of the following people push, hit, slap, kick, choke, or physically hurt you in any other way? For each person, check No if they did not hurt you during this time or yes if they did. My husband or partner | 1= NO  2= YES |
| PAD_XHUS | During your most recent pregnancy, did any of the following people push, hit, slap, kick, choke, or physically hurt you in any other way? For each person, check No if they did not hurt you during this time or yes if they did. My ex-husband or ex-partner | 1= NO  2= YES |
| PAD_OTH | During your most recent pregnancy, did any of the following people push, hit, slap, kick, choke, or physically hurt you in any other way? For each person, check No if they did not hurt you during this time or yes if they did. Someone else | 1= NO  2= YES |
